# Dental Education

**Published:** 1890-02

**Authors:** S. J. Andres

**Affiliations:** Montreal.


ARTICLE IV.

### DENTAL EDUCATION.

BY S. J. ANDRES, L. D. S., MONTREAL.

In looking back some years, the time was, when all that was required of a man to make him a dentist, was a certain amount of ingenuity, and deftness with tools; but that is among the things that were, and dentistry is no longer looked upon as a trade, but a profession, taking high rank with the learned professions of the present day, and will ere long be standing at the front, side by side with that of the medical profession itself. To attain that degree of high standing, it will require hard work on the part of the student, and a high grade of literary and scientific education.

There is no pursuit in life which does not require a certain amount of education and intellectual training. It is as equally necessary to the humblest artisan as to the most dignified profession. Any lack of it which the calling demands is at once discovered and brings with it regret and shame. The artisan who works in metal, wood or stone, and prepares it for the uses of civilization, in order to succeed in business, must possess it in a certain, but perhaps limited, degree; while the professional man who wishes to rise to eminence, who has the writings of great authors to " read, mark, learn and inwardly digest," to meet emergencies often of the most trying character, requiring sound judgment. . To give opinions which must stand the sever strain of public criticism, requires an education of the highest order.

To-day no one will doubt the statement that the man whose mind has been thoroughly trained in the attainment of a literary, scientific and classical education, is much better prepared to grasp and search out the hidden mysteries of professional lore, than one who has never had those advantages. The mind of an uneducated man is not able to cope with the facts and principles of science, and garner them up into the storehouse of his memory, " to bring forth fruit in good season," as seed sown upon fertile ground.

To fully recognize and appreciate relations that the teeth bear to the various organs of the body, it is necessary that the student should be familiar with its entire organism. Once it was thought that a perfect knowledge of the anatomy of the head was all that a dentist required in the practice of his profession, and there are many who even to-day are in practice holding the same opinion. That he should be familiar with that part of the body there can be no doubt, but that he should confine his knowledge to that portion of the human economy, and to it alone, is to be far behind in the progressive teachings of the dental schools and literature of the present day. It does not matter how thoroughly well up a man may be in the anatomy of the head, its bones and muscles, its blood vessels and nerves, which are distributed to the teeth, and as well as the microscopical structures of these organs, if the nervous system with its reflex action, the circulatory apparatus with its life-giving fluid, also the various other organs with their several functions, are unknown to him. It will be impossible for him to understand how the teeth can be the cause of cerebral and other sympathetic derangements that children are subject to during dentition, as well as the odontalgia of the parturient female, or the constitutional effects of carious teeth and vitiated oral secretions. Having been thoroughly trained in the knowledge of the whole human system, and qualified to diagnose with ease each case as it comes before him, he is able by judicious advice, proper remedies and well-ordered operations to give instant relief to his patient, whether child or adult; and when consulted by the intelligent medical practitioner (where he has reason to believe that the dental organs are in some way connected with the disease he is treating,) the dentist is qualified by his more familiar knowledge of their abnormal conditions, not only to be able to assist him in making his diagnosis, but brings credit to his profession and commands the respect of the physician by the knowledge shown.

The dental profession of to-day are largely responsible for the status of the profession of the future. If we are to be classed in the rank and file of those men who are called, to use a vulgar expression—"quacks," or "tooth carpenters"—and content to stay there, then we must fall to the level of the mechanic, and only require the education necessary for that purpose, but if we are to stand side by side with the members of the scientific and honorable profession of medicine, who are already willing to extend the right hand of fellowship to the men of our profession who will become entitled to it by being equal to them in educational ability and attainments, for that purpose, we require education of the highest order.

The highest object of the dental profession of to-day should be to elevate it to that high standard of excellence in education and respectability, which shall merit the recognition which we claim from our brethren of the medical profession.

I venture to make the assertion, that there is not one of the scientific professions of to-day that have made greater and more rapid strides in its advancement, than that of our own; that we shall not long be confined to the same narrow field of operations to which we have been in the past.

There are facial and oral diseases which properly come under the treatment of the dentist, many of them demanding the highest surgical skill and manipulation. And we have men in our profession who are equal to any occasion demanded of them. But there is room for more, and when the profession is supplied with the men qualified for the field that is open to them, then will they be called upon more generally to treat such cases.

I do not wish to be understood to decry the work of good men and true, of the past; for many of them have always been ahead of their times. Men who have given their time and energy as well as their money, to organize schools of dentistry, to give us dental literature of a high order, which is yearly improving and has been the means of putting our practice into its present scientific and practical status, have given to us a wider range of thought, and far better class of operations since their operations began and these schools have been organized.

Complaint has been frequently made (and perhaps not without some show of reason) that young men are graduated and sent out into the world who do not really possess the qualifications these institutions have claimed as their standard, and diplomas have been granted to persons wholly unfit to receive them. Are the colleges the only delinquents? Are not some of those who consider themselves members of the profession responsible for having sent out from their offices those who were equally unworthy of the title of dentist? Do they see to it that the parties they receive into their offices as students are possessed with brain sufficient to acquire the necessary educational qualifications, coupled with determined perseverance, to overcome the difficulties it requires to become a good practitioner? Are we both in precept and example without sin? Then let him that is without sin cast the first stone; at the same time remember that people who live in glass houses should not throw stones. Then let us cease from grumbling at the colleges; at this or that system of education, but remembering our own sins of omission, as well as theirs of commission, resolve that in the future any person desiring to enter the ranks of our profession shall possess the same qualifications in educational ability and requirements as are demanded by the best medical schools of our country. Instead of cavilling at the discrepancies of our schools, remember that we owe to them much praise for the good they have done. And if we desire them to be more exacting as to the qualifications of their graduates, mentally and morally, let us see to it that we be more exacting in our demands in the same direction of those we accept as students, and encourage by every way possible every legitimate means that can be used to improve and elevate the status of our membership.

Knowledge is power and to its possessor it gives intellectuality and efficiency; these attributes combined and used in the right direction will command respect in all lands and among all people, and every avocation where they are brought into action must assuredly rise in public esteem. We should not accept as students either in dental schools or in our offices any person who is not so qualified and whose mind is not fitted by education and a high sense of honor for the profession which he proposes to enter, so that he will guard well its interests, and with a faithful and honorable devotion to the calling he has chosen, do all that a true heart and an active hand can, to increase its efficiency and reputation.

It seems to be a current opinion that the profession dignifies the man. It is just the reverse. It is the man who dignifies it by shedding upon it the lustre of a well-trained and cultivated intellect; a well balanced mind and an unsullied character which the public will recognize and delight to honor; and I am glad that our profession is rapidly being filled with such men, and that legislation is being used with good effect to that end; that our legislators are recognizing the need of good laws well administered to carry on the good work and protect us from the direful influence of empiricism, and the public from imposition.

You have on this the 21st anniversary of this association organized an Odontological Society, if laid upon a solid foundation and well built upon, will be to the young men of this province who seek to enter the profession a means of incalculable benefit to them as a stimulant to help them on to the goal of perfection.

With a grand future before us, assisted by the laws we have succeeded in placing on our records as the corner stone of our society, let every licentiate feel that he is in honor bound to see that the laws shall be well administered and respected, and endeavor to build a structure upon them that shall be a monument of glory in the years that will come in memory of those who have helped to lay it; that shall shine out upon the pathway of the men who come after, guiding them toward and in the path of wisdom—as the beacon light on the rock-bound ocean shore guides the storm-tossed mariner to a peaceful harbor ; writing on our banner in letters of gold the word " Excelsior."—*Dominion Dental Journal.*

